# Influence of Equal Channel Angular Pressing Passes on the Microstructures and Tensile Properties of Mg-8Sn-6Zn-2Al Alloy

**DOI:** 10.3390/ma10070708

**Published:** 2017-06-27

**Authors:** Weili Cheng, Liang Tian, Shichao Ma, Yang Bai, Hongxia Wang

**Affiliations:** 1 School of Materials Science and Engineering, Taiyuan University of Technology, Taiyuan 030024, China; tianliang0108@link.tyut.edu.cn (L.T.); mashichao0125@link.tyut.edu.cn (S.M.); baiyang0087@link.tyut.edu.cn (Y.B.); wanghongxia@tyut.edu.cn (H.W.); 2 Shanxi Key Laboratory of Advanced Magnesium-Based Materials, Taiyuan University of Technology, Taiyuan 030024, China; 3 Key Laboratory of Interface Science and Engineering in Advanced Materials, Ministry of Education, Taiyuan University of Technology, Taiyuan 030024, China

**Keywords:** magnesium alloy, ECAP passes, microstructures, tensile properties

## Abstract

An I-phase containing Mg-8Sn-6Zn-2Al (wt %; TZA862) alloy was fabricated and subjected to different number of passes of equal channel angular pressing (ECAP) processing at 300 °C. The results showed that the alloys exhibited a bimodal microstructure, which consisted of fine dynamically recrystallized (DRX) grains and coarse non-DRX grains. When increasing the number of ECAP passes from 2 to 6, the fraction of DRX grains and the dispersed second phase particles subsequently increase. However, the fraction and particles then decrease once the number of ECAP passes increases to 8. After 6 ECAP passes, remarkable grain refinement was achieved and increasing the number of passes to 8 cannot further refine the microstructure. Furthermore, the alloys having undergone ECAP exhibited a strong ED-tilted texture, the intensity of which increased with an increase in the number of ECAP passes. The ultimate tensile strength (UTS; 338 MPa) and elongation (El.; 14.2%) of the alloy processed with 6 ECAP passes were considerably higher compared to those of the other materials that had undergone ECAP. These significant enhancements were attributed to extensive grain boundary strengthening, precipitation strengthening and a higher work-hardening capacity.

## 1. Introduction

The Mg–Sn alloy system with Mg_2_Sn particles that have a high melting temperature (*T*_m_ = 770 °C) is comparable to the Mg–RE system containing thermally stable precipitates (*T*_m_ = approximately 500–750 °C) in term of strength and formability [1,2,3,4,5]. Furthermore, previous studies have shown that Mg–Sn based alloys exhibit a greater strength than commercially extruded Mg–Al based alloys under the same testing conditions [6,7]. However, a previous investigation [8] has indicated that coarse and fine particles within the α-Mg matrix typically coexist in the Mg–Sn alloy system. Thus, the way to refine the Mg_2_Sn precipitates and introduce a new strengthening particle to enhance the mechanical properties is of particular importance. Recently, Kim et al. [9] reported that the icosahedral quasicrystal phase (I-phase) could be obtained in Mg–Sn based alloy by selecting a suitable Zn/Al mass ratio. Furthermore, they found that the tensile properties of I-phase-bearing alloys at room temperature were significantly improved after hot rolling through dynamic precipitation strengthening [9]. Our previous study [10] has shown that I-phase could be formed in a TZA862 alloy and thus, it was chosen for studying the crystallographic relationship between the I-phase and Mg_2_Sn precipitates during ECAP processing.

As one of the most effective severe plastic deformation (SPD) techniques, ECAP provides an effective way to refine grain size and secondary phases by inducing a large shear deformation [11]. On the other hand, the ECAP process can result in a simple shear being applied to the materials at the channel angled at 90° and therefore, texture modification can be achieved by changing the orientation of the applied stress [12,13]. It is well known that the number of ECAP passes has an important influence on the microstructure and mechanical properties of Mg alloys. Lapovok et al. [14] reported that greater strengthening could be achieved with more than four ECAP passes in the Mg-4Zn-4Y (wt %) alloy. Recently, Liu et al. [15] have reported that the strength and ductility of Mg-8Y-4Zn (wt %) alloy could be improved effectively by a 6-pass ECAP. Meanwhile, Ma et al. [16] showed that the ultimate tensile strength (UTS) and yield strength (YS) of a sample that had undergone a 32-pass ECAP had no obvious enhancement when compared with those of the Mg-4.9Zn-1.4RE-0.7Zr (wt %) alloy that had undergone an 8-pass ECAP. Thus, in order to achieve a combination of good mechanical properties and lower cost for industrial applications, a maximum of 8 ECAP passes are chosen.

The number of ECAP passes plays a crucial role, similar to extrusion temperature and speed, in determining dynamic precipitation and recrystallization behaviors during ECAP processing and resultant mechanical properties [11]. As mentioned above, the strengthening of different alloy systems could be obtained by implementing different numbers of ECAP passes, indicating that grain size, recrystallization fraction, precipitate phase particles and texture were greatly affected by the number of ECAP passes. Despite all this, there is still a degree of uncertainty in the effect of number of ECAP passes on the microstructure characteristics and mechanical properties of I-phase containing Mg–Sn based alloy system. Therefore, the present work aims to discuss the role of the number of ECAP passes on the Mg–Sn–Zn–Al system containing I-phase that has undergone ECAP in order to pave the way for development of the alloys. By studying this, it might possibly reveal the dependence of the strengthening effect of the grain boundary and I-phase as well as Mg_2_Sn precipitates on the number of ECAP passes in the TZA862 alloy. This study will be helpful for the further development of the high-strength and low-cost Mg alloys.

## 2. Experimental Procedure

Ingot with an actual composition of Mg-7.89Sn-5.93Zn-1.94Al (wt %, TZA862) alloy was prepared from the elements Mg, Sn, Zn and Al with 99.99% purity by melting via electric resistance under the protection of a mixed atmosphere of CO_2_ and SF_6_. The molten alloy was poured at 720 °C into a preheated steel mold (approximately 200 °C). Solid solution treatment (SST) was carried out at 320 °C for 3 h and then 450 °C for 20 h, followed by water-quenching. Cuboid samples with a dimension of 10 mm × 10 mm × 55 mm prepared for ECAP processing were cut off from the central part of the SST ingot. The ECAP process was carried out at 300 °C with an average extrusion speed of 10 mm/min using a die with *φ* = 90° and *ψ* = 20° (*φ*: inner arc of curvature; *ψ*: outer arc of curvature) following the route Bc (the specimen is rotated by 90° in the same direction between each pass). The alloys subjected to 2-, 4-, 6- and 8-pass ECAP were named the 2p, 4p, 6p and 8p samples, respectively.

The tensile properties of the samples that had undergone ECAP were measured by a DNS100 electronic testing machine (SFMIT Ltd. Changzhou, China) at the initial tensile strain rate of 1 × 10^−3^ s^−1^ at room temperature. The microstructural characteristics of the specimens were observed using an optical microscope (OM, Leica DM2700M, Leica Microsystem GmbH, Wetzlar Germany), a scanning electron microscope (SEM, TESCAN MIRA3, TESCAN Ltd., Brno-Kohoutovice, Czech Republic) and a transmission electron microscope (TEM, JEM-2100F, JEOL Ltd., Tokyo, Japan). The alloy phases were analyzed by X-ray diffraction (XRD, Cu-Kα, Y-2000, Dandong Ray Instrument Co., Ltd., Dandong, China), which was combined with selected area diffraction patterns. The polished surfaces of metallographic samples were etched in a solution of 3 g of picric acid, 10 mL of acetic acid, 10 mL of H_2_O and 50 mL of ethanol. The average grain sizes (number fraction) and the fractions of DRX grains and precipitates (area fraction) were calculated using three optical and/or SEM micrographs by the software Image-Pro plus 6.0 (Ipwin32, Media Cybemetics Co., Rockville, MD, USA).

## 3. Results and Discussion

### 3.1. Microstructure

**Figure 1:**
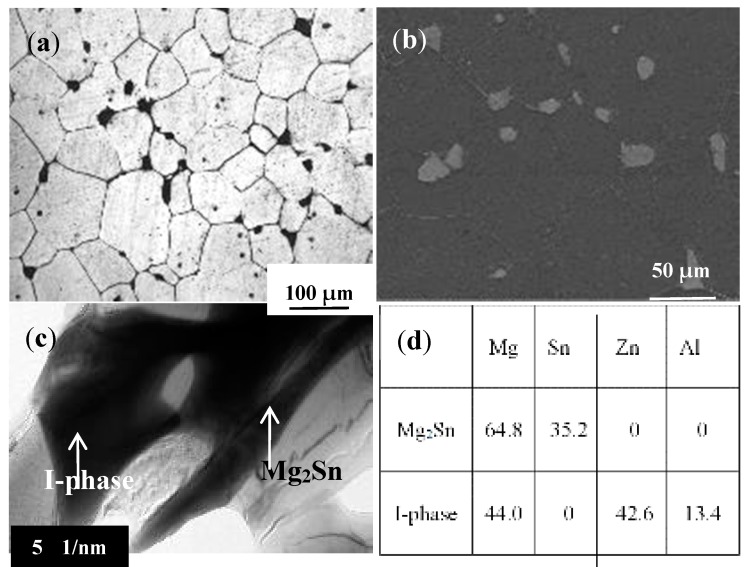
The microstructure of the solid solution treatment (SST) TZA862 alloy: (**a**) optical microscope (OM); (**b**) scanning electron microscope (SEM); (**c**) transmission electron microscope (TEM) and (**d**) the energy dispersive spectroscopy (EDS) result (by %) of the I-phase and Mg_2_Sn in (**c**).

As shown in Figure 1a,b, the SST alloy exhibited equiaxed grains with an average grain size of 73.9 µm and a quantity of remaining coarse particles. In addition, the microstructure revealed by TEM (Figure 1c) clearly indicated the presence of two different particles types, which were coexisting with each other. From the energy dispersive spectroscopy (EDS) results in TEM, the particles could be determined as Mg_44.0_Zn_42.6_Al_13.4_ and Mg_2_Sn, respectively. Similar to a previous report [10], the Mg_44.0_Zn_42.6_Al_13.4_ phase could be marked as I-phase.

**Figure 2:**
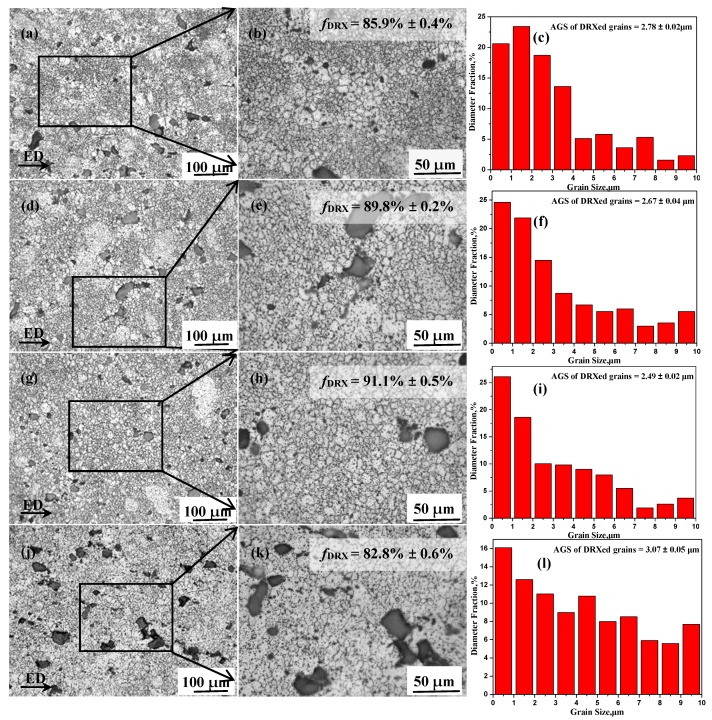
Optical images and grain size distribution of TZA862 alloys with different numbers of equal channel angular pressing (ECAP) passes having been conducted: (**a**–**c**) 2p; (**d**–**f**) 4p; (**g**–**i**) 6p and (**j**–**l**) 8p. ED represents the ECAP direction.

The images of the microstructure and grain size distribution map of TZA862 alloys conducted with different numbers of ECAP passes are shown in Figure 2, while the related microstructural characteristics are also summarized in Table 1. The samples that underwent ECAP exhibit a partially recrystallized structure with a bimodal grain size distribution. The relative size of the coarse grains is approximately 10 µm, while a considerable fraction of the grains is less than 2 µm. In addition, the fraction of dynamic recrystallized grains increased from 87.2% (2p) to 91.1% (6p), before it decreased to 82.8% (8p). Moreover, the 6p sample exhibited the smallest average DRX grain size (AGS) with a size of 2.49 µm, while the AGS of the 8p sample was increased to 3.07 µm. This was related to the different suppression degrees of grain growth by the Zener drag of the secondary phase particles [8]. In general, the variation in the grain size and DRX fraction (*f*_DRX_) during hot deformation are closely related to the fraction, size and morphology of secondary phase particles [6,7,8] and thus, the dependence of *f*_DRX_ on secondary phase particles will be discussed in Section 3.2.

**Figure 3:**
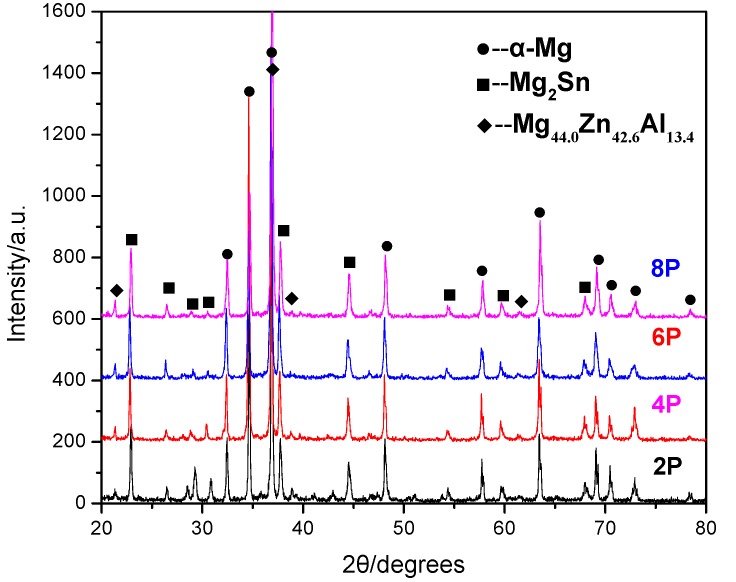
The XRD patterns of the TZA862 alloys after different numbers of ECAP passes.

The XRD patterns of the TZA862 alloys processed with different numbers of ECAP passes are shown in Figure 3. All the patterns exhibit three sets of peaks: α-Mg, Mg_2_Sn and I-phase (Mg_44.0_Zn_42.6_Al_13.4_). It was noted that, with an increase in the number of ECAP passes from 2 to 6, the peaks of Mg_2_Sn and I-phase gradually increased due to the enhanced dynamic precipitation during the ECAP process. The peaks of Mg_2_Sn and I-phase in the 8p sample are almost similar to those in the 6p one, indicating that the amount of the secondary phase particles remains nearly constant in the 6p and 8p samples. It implied that particles redistribution or crushing of Mg_2_Sn and I-phase particles during the ECAP process could be dominant, while dynamic precipitation of the particles was negligible when the number of ECAP passes increased from 6 to 8.

### 3.2. Precipitates

**Figure 4:**
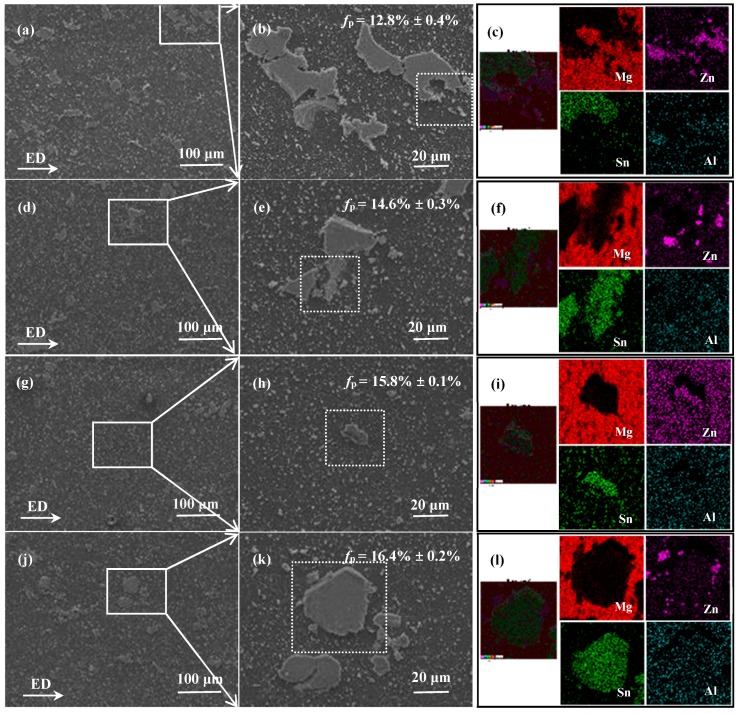
The SEM image and surface SEM–EDS results of TZA862 alloy with different numbers of ECAP passes having been conducted: (**a**–**c**) 2p; (**d**–**f**) 4p; (**g**–**i**) 6p and (**j**–**l**) 8p. EDS mapping was conducted in the area circled by the dotted line in Figure 4 (**b**, **e**, **h** and **k**).

Figure 4 shows the SEM images of the 2p, 4p, 6p and 8p samples. As shown, there are four types of precipitates: the precipitated nano-scale particles, the micro-scale particles crushed during the ECAP progress, the non-deformed particles (with sizes remaining consistent with those of coarse particles in SST sample) and the redistributed particles (larger than the coarse particles in the SST sample). From measurements, the fractions of secondary phase particles in the 2p, 4p, 6p and 8p samples are 12.8%, 14.6%, 15.8% and 16.3%, respectively. The increment in the fraction of secondary phase particles is primarily due to the enhanced dynamic precipitation with an increase in the number of ECAP passes. Additionally, it can be determined that the fraction of dispersed fine particles along the boundaries and inside the grains in the 6p sample is relatively large. It was noted that a number of coarse redistributed and non-deformed particles can be found in the studied samples. These particles are large enough to cause a serious stress concentration and provide sites for fracture initiation [17], which is detrimental to ductility. Meanwhile, the fraction of the coarse particles in 2p, 4p and 8p samples are relatively high compared to that of the 6p specimen, implying relatively poor ductility of 2p, 4p and 8p samples.

The surface SEM–EDS results validated the formation of the I-phase and Mg_2_Sn phases, with the I-phase coexisting with the Mg_2_Sn. A similar phenomenon could also be found in the Mg-Zn-RE alloy [18] as the I-phase coexisted with the W-phase (Mg_3_Zn_3_RE_2_), which is closely related to the micro-area composition. Similarly, in the present research, the zones around the coarse Mg_2_Sn particles are poor in Sn but rich in Zn and Al, which meets the composition requirements of I-phase formation. Therefore, I-phase is preferentially formed in these areas. However, the correlation between Mg_2_Sn particles and I-phase formation is not well understood at present and detailed analyses are currently being conducted.

**Figure 5:**
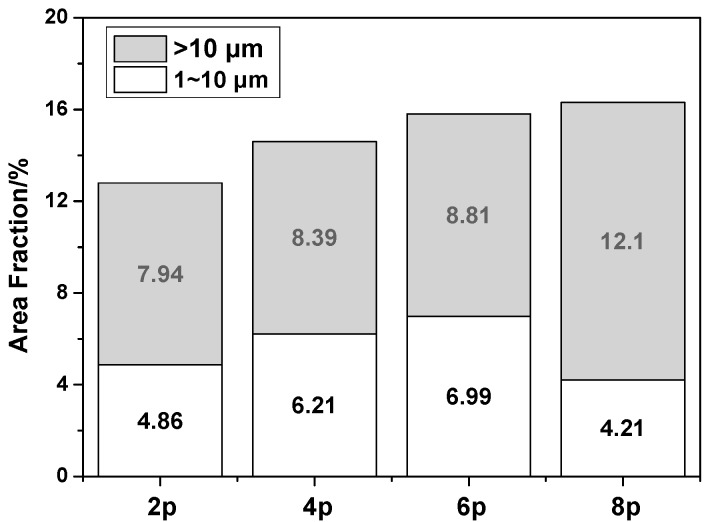
Particle-size distribution in the area fraction.

The particle-size distribution in the area fraction is shown in Figure 5. As shown, the 8p sample has the largest fraction of particles (*f*_p_ = 16.3%), with the coarse non-deformed particles and redistributed particles accounting for a very large proportion (74.2%). In comparison, the fraction of relatively fine precipitated particles (1–10 µm) is as low as 4.21%. However, the 6p sample has the largest number of particles having a size of 1–10 µm (*f*_p_ = 6.99%). Several publications have shown that particles with a size of 1–10 µm can act as nucleation sites for DRX during hot deformation, because a higher dislocation density and large orientation gradient can be induced at the deformed zones in the vicinity of the particles, which is the so-called particle stimulated nucleation (PSN) effect [19]. For this reason, a relatively small fraction of 1–10 µm particles in the 8p sample results in a relatively weak PSN effect, which led to a lower DRX fraction after ECAP (82.8%), As for 6p sample, the highest DRX fraction (91.1%) is considerable dependent on the highest fraction of the 1–10 µm particles.

**Figure 6:**
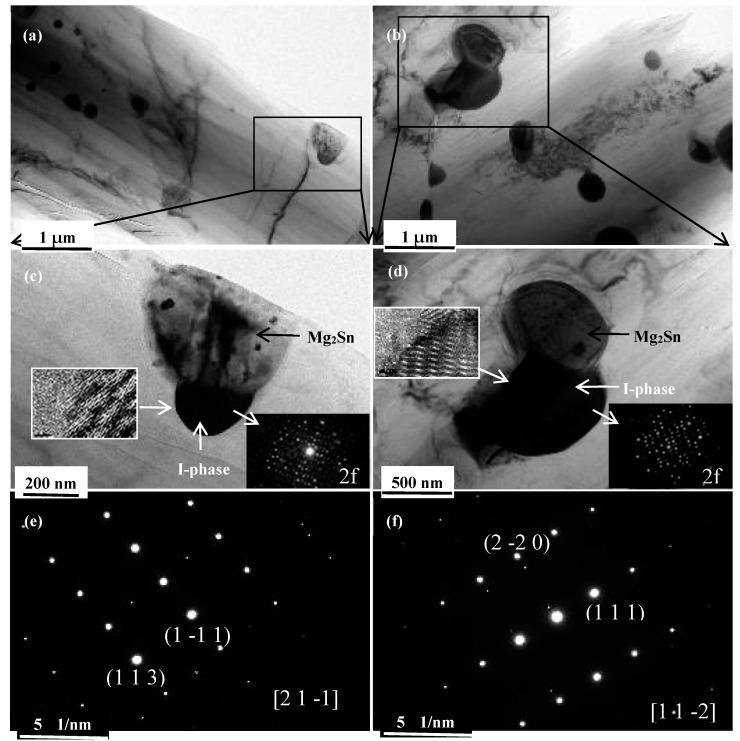
The TEM images of the 6p and 8p sample. Bright field images: (**a**,**c**) 6p sample and (**b**,**d**) 8p sample (inset: the SADP of I-phase and the HRTEM of the interface between the I-phase and the surrounding α-Mg matrix); SADP of the Mg_2_Sn phase: (**e**) 6p sample and (**f**) 8p sample. SADP: Selected Area Diffraction Patterns; HRTEM: high-resolution transmission electron microscopy.

To distinguish the nano-scale precipitates more clearly and describe the crystallographic relationship between the I-phase and Mg_2_Sn phases, TEM observations were carried out on the 6p and 8p samples. As indicated in Figure 6, there are two types of nano-scale precipitates with different contrasts. From the selected area electron diffraction (SAED) results, it was determined that the I-phase exhibited a greater contrast compared with the Mg_2_Sn phase. Furthermore, it is interesting to find out that most of the I-phases are located between the α-Mg and Mg_2_Sn phases, indicating that the formation of I-phase lags behind that of the two eutectic phases during solidification [20]. The average size of precipitates in the 8p sample is significantly larger than that of the 6p sample. In particular, the difference between the size of the I-phase in 6p and 8p samples should also be taken into account. The average size of the I-phase in the 6p sample is approximately 150 nm, while the corresponding value increased to be above 500 nm in the 8p sample. As indicated in Figure 6c,d, the I-phase in the 6p and 8p sample is oriented along the 2-fold (2f) rotational symmetry axis. When combined with the SADP (Selected Area Diffraction Patterns) of the Mg_2_Sn phase (Figure 6e,f), the orientation relationship between the I-phase and the connected Mg_2_Sn phase in 6p and 8p can be expressed as [2f]//[2 1 −1]_Mg2Sn_ and [2f]//[1 1 −2]_Mg2Sn_. In addition, the high-resolution transmission electron microscopy (HRTEM) images of the interface between the I-phase and the surrounding α-Mg matrix in the 6p and 8p sample (inset in Figure 6c,d) show that the interfaces are both semi-coherent interfaces, which can inhibit crack initiation and provide an enhanced strengthening effect [21].

### 3.3. Texture

**Figure 7:**
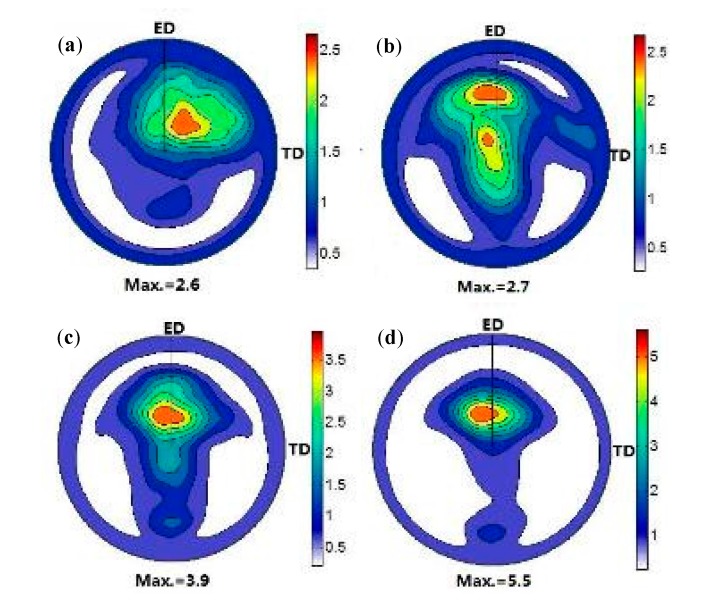
The (0002) pole figures of the of TZA862 alloys with different numbers of ECAP passes having been conducted: (**a**) 2p; (**b**) 4p; (**c**) 6p and (**d**) 8p.

The (0002) pole figures of TZA862 alloys subjected to different numbers of ECAP passes are shown in Figure 7. It can be seen that all the Mg alloys that had undergone ECAP exhibit an ED-tilted texture and the maximum intensity is not located in the center of the (0002) pole, which means that the c-axis of most grains is tilted from TD/ND to the extrusion direction (ED) after ECAP. In addition, the intensity of the tilted texture increases with a gradual increase in the number of ECAP passes. The maximum intensity of the (0002) basal pole increases remarkably in the 8p sample, indicating that ECAP passes have an important effect on the reinforcement of texture on TZA862 alloy. As indicated, the distribution of the (0002) pole figure became more and more concentrated and the c-axis of grains almost is orientated on the ND–ED plane with an increase in the number of ECAP passes. This is one of the reasons why the maximum intensity increased gradually from 2.6 to 5.5 with an increase in the number of ECAP passes from 2 to 8. Furthermore, a previous study indicated the DRX grains form from the randomly oriented grains. Thus, a larger DRX fraction will increase the randomness of the grain orientation and finally decrease the texture intensity [22]. This is the main reason for the 8p sample with the lowest DRX fraction exhibiting the highest texture intensity. In addition, it is reported that the existence of coarse non-deformed particles will weaken the basal plane texture [23]. Thus, the presence of coarse non-deformed particles in alloys undergoing ECAP will weaken the texture intensity to a certain degree. Under the influence of contrary factors between the DRX fraction and coarse non-deformed particles, the texture intensity increased with an increase in the number of ECAP passes, indicating that the DRX fraction is dominant in controlling the texture evolution. The aforementioned texture characteristics in the 8p sample will be beneficial to the strength but will be detrimental to the El. [24] when an applied stress is parallel or perpendicular to the basal plane.

### 3.4. Tensile Properties

**Figure 8:**
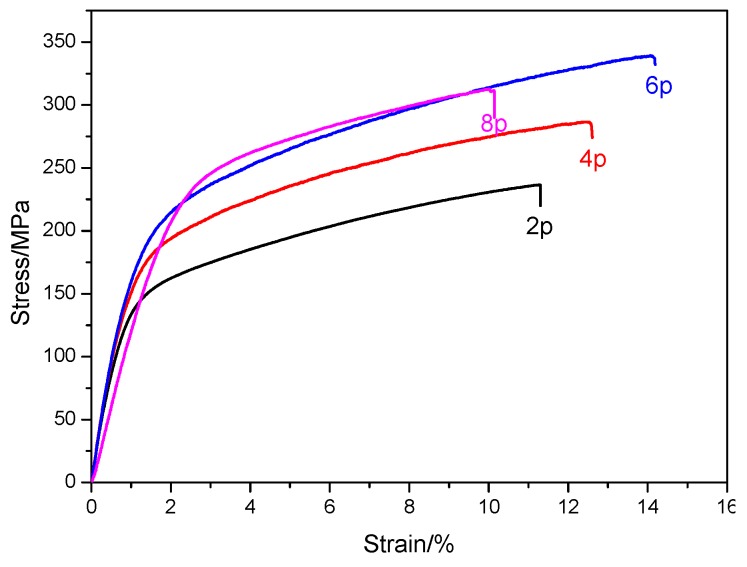
Typical tensile stress-strain curves of the samples that had undergone ECAP at room temperature.

The room temperature tensile properties of samples with different numbers of ECAP passes conducted are shown in Figure 8 and summarized in Table 1. It is noted that the peak point (failure to fracture) of the stress-strain curve was used as UTS and the ratio of displacement of the tension rod and gage length as El., respectively. It can be seen that the yield strength (YS) increased from 161 to 235 MPa with an increase in the number of ECAP passes from 2 to 8. However, the ultimate tensile strength (UTS) and El. increased to their maximum values in the 6p sample. The difference between UTS and YS obtained in a tensile test can be used to define the work-hardening capacity (HC = (UTS–YS)/YS), which is associated with the grain size based on the Hall-Petch relationship [25]. The values are listed in Table 1. As seen, the work-hardening capacity of the samples that underwent ECAP increases to a peak value of 0.67 in the 6p sample and decreases to a minimum value of 0.38 in the 8p sample.

**Figure 9:**
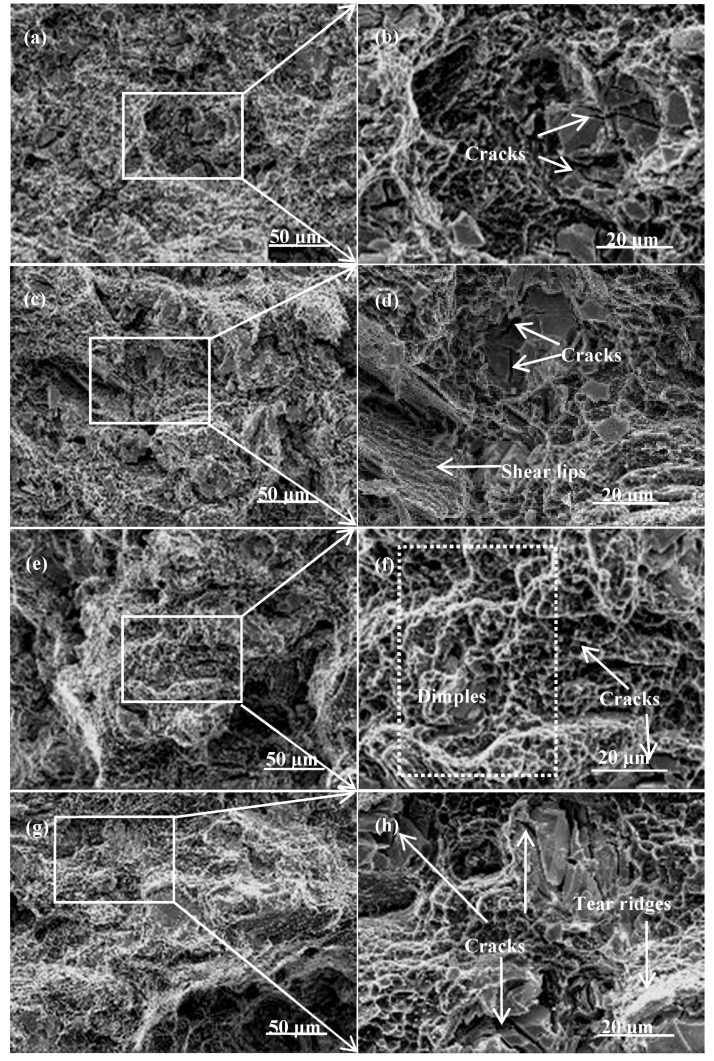
SEM fractography of the alloys having undergone ECAP: (**a**,**b**) 2p; (**c**,**d**) 4p; (**e**,**f**) 6p and (**g**,**h**) 8p.

The SEM fractography of the samples that underwent ECAP is shown in Figure 9. As exhibited, many dimples, cleavage planes, shear lips, tear ridges and particles cracking could be observed on the fracture surfaces, suggesting a mixed fracture mode of ductile–brittle type. On the one hand, a number of particles cracking and tear ridges can be obviously observed in the 2p, 4p and 8p samples, indicating the relatively poor ductility. On the other hand, in the case of the 6p sample, a large number of fine dimples separated by sharp tear ridges and cracking becomes more obvious, providing clear evidence for its larger El. compared to that of other samples.

**Figure 10:**
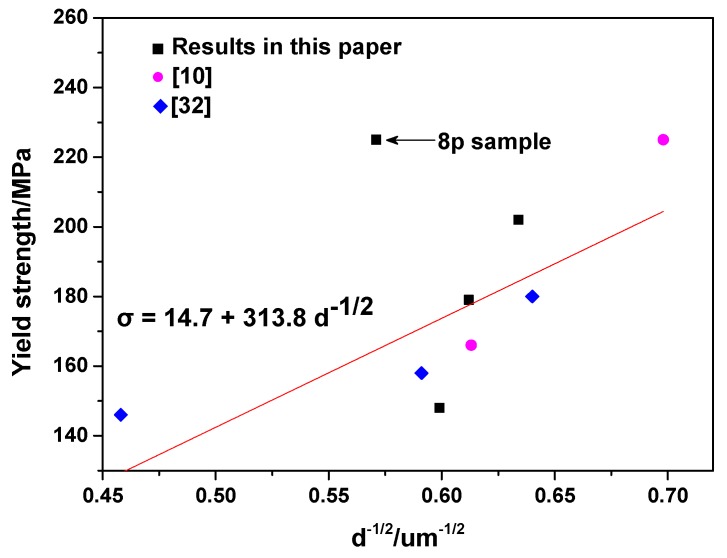
Hall-Petch relation of the Mg–Sn alloy having undergone ECAP as calculated from this paper and our previous results (reference [10,32]).

The strength of the polycrystalline Mg alloys is strongly influenced by grain size, second phase particles and crystallographic texture [26,27,28,29]. It is noted that the tensile properties of the textured Mg alloys are not recognized by the conventional Hall-Petch equation, because they are instead determined by the combined effects of grain size and crystallographic texture [30]. In general, the constant term (*σ*_0_) and slope (*k*) in the Hall-Petch relationship can be influenced by the variation of the texture intensity in Mg alloys [31]. Thus, in order to comprehensively considering the combined effect, based on our previous work [10,32] and the present research, an amendatory Hall-Petch equation suitable for the Mg–Sn–Zn–Al alloys that undergone ECAP with a maximum texture intensity of less than 4 was established as following: *σ* = 313.8·*d*^−1/2^ + 14.7. The results are shown in Figure 10. According to the above equation, the increment of YS due to grain refinement from 2.78 µm (2p sample) to 2.49 µm (6p sample) is calculated as 10.5 MPa. Secondly, references [6,33] reported that uniformly distributed fine particles could improve the strength of an alloy by hindering the movement of mobile dislocations during the extrusion process. Moreover, according to the theory proposed by Gladman et al. [34], a smaller size and larger fraction of precipitates will lead to better precipitation strengthening. Thus, a higher area fraction of precipitates, which is 15.8%, in the 6p sample is considered to possibly lead to better strengthening. Finally, it is interesting to find that the point in Figure 10 representing the 8p sample (maximum texture intensity being of 5.5) deviates from the linear fitting results between the grain size and the YS. In essence, the relationship between YS and grain size in the 8p sample cannot be expressed by the present calculated Hall-Petch relationship. Similarly, Yuan et al. [35] found that the highly textured AZ31 alloy can be strengthened by the texture effect due to the texture intensity being proportional to the constant term (*σ*_0_) and slope (*k*) in the Hall-Petch relationship in Mg alloys. Furthermore, many researchers [26,31,36] have also been revealed that the modified texture could improve the strength of hexagonal close packed (HCP) polycrystals through its effect on the variables of the Hall-Petch equation. Thus, the reason for 8p sample not being able to be expressed by the present calculated equation may be related to its relatively high texture intensity. It is noted that the 8p sample has the highest YS among the samples, with the average grain size being the largest (3.07 µm) and the fraction of the fine precipitates (1–10 µm) being the lowest (4.21%). This indicated that the grain boundary strengthening and precipitation strengthening are not beneficial in improving YS. As a consequence, the highest YS of the 8p sample mainly resulted from the texture strengthening as shown in other highly textured Mg alloys.

Note that, the El. of the 6p sample is relatively high compared to other samples, which is different from the typical relationship between strength and ductility [37]. First, the critical resolved shear stress (CRSS) of the non-basal slip system in the fine-grained polycrystalline Mg would decrease to the level of the basal slip due to the enhanced grain-boundary compatibility [38], which is favorable in improving the ductility. In addition, it is well known that a large work-hardening capacity leads to a low sensitivity to strain localization, resulting in a greater El. [39]. This indicates that the 6p sample has the lowest sensitivity to strain localization, as it resulted in a higher El. Moreover, it has been reported that the non-deformed particles are large enough to cause a high-stress concentration due to the pile-up of mobile dislocations during plastic deformation [10]. The combined effect of grain size, Hc value and area fraction of non-deformed particles resulted in the present El. values of the studied specimens.

## 4. Conclusions

(1) I-phase (Mg_44.0_Zn_43.7_Al_12.3_), coexisting with Mg_2_Sn particles, was found in both SST and TZA862 alloys that had undergone ECAP.
(2) The samples that had undergone ECAP exhibited a partially recrystallized structure and a strong ED-tilted texture. The largest fraction (91.1%) and finest average size (2.49 µm) of DRX grains were obtained in the 6p sample. In addition, the maximal texture intensity increased from 2.6 to 5.5 with as increase in the number of ECAP passes from 2 to 8.
(3) The enhanced tensile properties of the 6p sample (YS = 202 MPa, El. = 14.2%) were mainly related to the grain boundary strengthening and precipitate strengthening as well as the high work-hardening capacity (Hc = 0.67).
(4) The YS of the 8p sample (225 MPa) had a strong dependence on the material texture, while the lowest El. among the tested samples (10.1%) was strongly related to the largest amount of the coarse particles and smallest Hc (0.39).

## Figures and Tables

**Table 1:** The microstructural characteristics and tensile properties of the alloys that underwent equal channel angular pressing (ECAP).

Conditions	Microstructural Characteristics				Tensile Properties			
	*f*_DRX_ (%)	*d*_DRX_ (µm)	*f*_p_ (%)	Texture Intensity	UTS (MPa)	YS (MPa)	El. (%)	Hc = (UTS–YS)/YS
2p	85.9 ± 0.4	2.78 ± 0.02	12.8 ± 0.4	2.6	217 ± 8	148 ± 5	11.2 ± 0.3	0.47 ± 0.02
4p	89.8 ± 0.2	2.67 ± 0.04	14.6 ± 0.3	2.7	286 ± 6	179 ± 3	12.6 ± 0.4	0.60 ± 0.03
6p	91.1 ± 0.5	2.49 ± 0.02	15.8 ± 0.1	3.9	338 ± 5	202 ± 6	14.2 ± 0.2	0.67 ± 0.01
8p	82.8 ± 0.6	3.07 ± 0.05	16.4 ± 0.2	5.5	313 ± 7	225 ± 3	10.1 ± 0.3	0.39 ± 0.02

Note: DRX: dynamically recrystallized; UTS: ultimate tensile strength; YS: yield strength; EL.: elongation.
